# The Expression Pattern of PLIN2 in Differentiated Adipocytes from Qinchuan Cattle Analysis of Its Protein Structure and Interaction with CGI-58

**DOI:** 10.3390/ijms19051336

**Published:** 2018-05-01

**Authors:** Peiwei Li, Yaning Wang, Le Zhang, Yue Ning, Linsen Zan

**Affiliations:** 1 College of Animal Science &Technology, Northwest A&F University, Yangling 712100, Shaanxi, China; lipeiweilpw@163.com (P.L.); wangyn1992@gmail.com (Y.W.); yuezhangzi@163.com (L.Z.); ningyueny@163.com (Y.N.); 2 National Beef Cattle Improvement Center, Yangling 712100, Shaanxi, China

**Keywords:** PLIN2, expression pattern, bovine adipocytes, protein structure, Protein–Protein interaction, mutation model

## Abstract

PLIN2 (Perilipin-2) is a protein that can anchor on the membrane of lipid droplets (LDs), playing a vital role in the early formation of LDs and in the regulation of LD metabolism in many types of cells. However, little research has been conducted in cattle adipocytes. In the present study, we found that the expression of PLIN2 mRNA peaks at Day 2 during cattle adipocyte differentiation (*p* < 0.01), but PLIN2 protein levels maintain high abundance until Day 4 and then decrease sharply. We first built an interaction model using PyMOL. The results of a pull-down assay indicated that bovine PLIN2 and CGI-58 (ABHD5, α/β hydrolase domain-containing protein 5) had an interaction relationship. Furthermore, Bimolecular Fluorescence Complementation-Flow Cytometry (BiFC-FC) was used to explore the function of the PLIN2-CGI-58 interaction. Interestingly, we found that different combined models had different levels of fluorescence intensity; specifically, PLIN2-VN173+CGI-58-VC155 expressed in bovine adipocytes exhibited the highest level of fluorescence intensity. Our findings elucidate the PLIN2 expression pattern in cattle adipocytes, the protein structure and the function of protein–protein interactions (PPI) as well as highlight the characteristics of bovine PLIN2 during the early formation and accumulation of lipid droplets.

## 1. Introduction

Lipid droplets (LD), which are generally called vesicles, have a structure similar to small balloons that are full of neutral lipids and anchored by many different proteins [1,2]. Although LDs are particularly abundant in adipocytes in mammals, they have recently been recognized to be ubiquitous organelles that exist in most types of eukaryotic cells [3]. LD plays a vital role in many cell functions, such as energy storage, metabolism, and signal transduction [4,5]. However, there has been little research conducted on the fundamental mechanisms of LD biogenesis and lipid metabolism in LDs.

An LD is surrounded by a single layer of phospholipid molecules [6], the core components of which consist of hydrophobic neutral fats (primarily ATGL (adipose triglyceride lipase) and cholesterol ester) that are maintained on the surface with specially structured anchor proteins [7]. Proteins that are recruited by LDs mediate many metabolic functions, including phosphatidylcholine and triglyceride synthesis [8]. The PAT (Perilipin/ADRP/TIP47) family members, including PLIN1 (perilipin-1)/perilipin, PLIN2/ADRP, PLIN3/tail-interacting 47kDa protein, PLIN4/S3–12, and PLIN5/ myocardial LD protein (MLDP), play pivotal roles in the formation and/or degradation of LDs [5,9], and this family of proteins not only reveals TAG (triacylglycerol) packaging but also order packaging by managing the droplet interface with the cytosol [10]. The PAT family is widespread, occurring in different species and playing a role in the process of lipid droplet accumulation in other types of cells [5]. The stimulation of lipid droplet formation in cells incubated with acetylated, low-density lipoprotein leads to the clustering of PAT family proteins in raised plasma membrane domains [6]. PLIN2 was originally found in fat and steroid-generated cells. A recent study also showed that PLIN2 is a type of factor primarily expressed in the early process of adipocyte differentiation and promotes fat droplet formation in muscle cells [11]. It is important to note that, during the early stages of ovulation in mouse oocytes, PLIN2 expression levels show an increasing trend, which might suggest that it is involved in the maintenance of the lipid stocks that are necessary to support embryo development following the fertilization of IVM (in vitro-matured) oocytes [12]. Studies on 3T3-L1 murine adipocytes showed the preferential association of PLIN2 with lipid droplets compared with PLIN1, implying that PLIN2 is more important than PLIN1 in lipid formation [13], which indicates that PLIN2 can be used as a marker gene for precursors during adipocyte differentiation. Thus, PLIN2 has important physiological significance.

PLIN2 has been shown to be upregulated in mouse oocytes after preovulatory hormonal stimulation [14]. PLIN2 interacts with lipid droplets through a hydrophobic mechanism to gather phospholipids around a neutral lipid core, which causes each lipid droplet to form around a monolayer. Studies in mice have shown that PLIN2 is usually located on small droplets [15]. These results lead us to question whether there are different expression patterns for the gene and the protein in live stocks, what the structure of this protein is, and whether this protein could be involved in interactions with other proteins. Some studies have shown that CGI-58 (Comparative Gene Identification-58) is part of a complex that activates a co-factor for ATGL (Adipose Triglyceride Lipase) [16] by combining with perilipin [17], and CGI-58 plays an important but poorly understood role in triacylglycerol catabolism [16,18]. Of note, some studies have found that, during the differentiation stage, the PLIN2 protein was degraded via the ubiquitin-proteasome system [19]. Many proteins are subjected to the system, and its dysregulation is linked to the pathogenesis of diseases, such as cancer and neurodegeneration. Moreover, PLIN2 is degraded in the cytosol in an N-terminal-sequence-dependent manner and becomes stabilized when localized on LDs [20].

We have identified more than 100,000 protein sequences [21], but the folding and processing mechanisms that determine the secondary and tertiary structures remain largely unknown. The structure of nearly a thousand proteins is currently known, due to their size and the involved simulation timescales, however, it is only recently that simulations have enabled the prediction of biological properties [22]. For membrane proteins, only some hundreds of structures are known, due to the highly hydrophobic and/or amphipathic properties of membrane proteins, which also makes it difficult to produce a homogeneous, stable, and pure sample for use in structural studies [21,23]. There are three types of membrane proteins: peripheral membrane proteins, integral membrane proteins, and lipid-anchored proteins. Moreover, the structures of lipid-anchored proteins are more difficult to resolve due to their strong hydrophobicity and irregular adhesion.

Little research on the bovine *Plin2* gene has been performed, as most research has focused on animal models and humans. Based on its role in adipogenesis, which was demonstrated in mice, we also propose the hypothesis that similar variations in the PLIN2 mRNA and protein expression pattern exist in cattle. Because of the expression patterns, we assumed that this gene might affect the meat quality traits in cattle. Hence, we focused on Qinchuan cattle, a well-known indigenous cattle breed found in central China, for which improvements in the growth rate and the quality of meat production, such as marbling, are needed [24] to produce high-class beef and to became more popular with customers. Considering the importance of PLIN2 function for LD formation, we also determined the molecular characterization of bovine PLIN2 by using bioinformatics. Screening for genetic variations was performed by direct sequencing, and the protein structure was modeled. Moreover, we constructed different interaction models to determine its efficiency. Our results are potentially beneficial for further research in adipocyte differentiation in beef cattle. Additionally, this study is the first to identify PLIN2 expression in cattle adipocytes and to characterize an interaction function in vitro and in vivo.

## 2. Results

### 2.1. Sequence Homology, Inferred Phylogenetic Tree and Sequence Alignments

**Figure 1:**
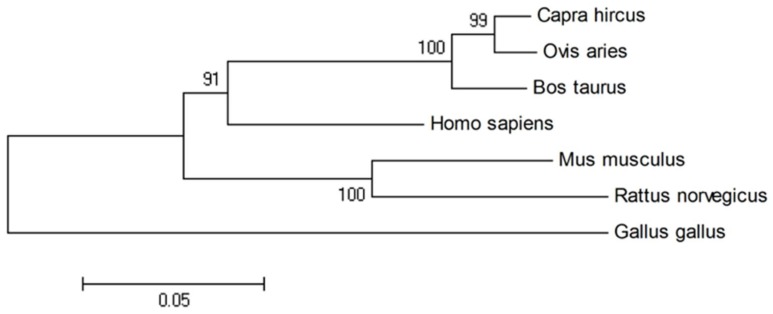
Phylogenetic tree for amino acid sequences of the *Plin2* gene in seven species, with bootstrap confidence values at the branch nodes. Branch lengths indicate the evolutionary distances. *Bos taurus* (NP_776405.1) with other vertebrates, such as *Homo sapiens* (NP_001113.2), *Rattus norvegicus* (NP_001007145.1), *Mus musculus* (NP_031434.3), *Gallus* (NP_001026591.1), *Capra hircus* (NP_001272525.1) and *Ovis aries* (NP_001098402.1).

The *Plin2* gene map on bovine chromosome 8 consists of nine exons and encodes 451 amino acid resides. BLAST analysis revealed that the amino acid sequence of bovine PLIN2 shared a high similarity with other species, with the following levels of identity: human (82%), rat (73%), mouse (75%), chicken (43%), goat (95%) and sheep (96%). We could also demonstrate that among *Bovidae* (bovine, goat, and sheep) the PLIN2 protein was more conserved compared with other species. The phylogenetic tree (Figure 1) shows the relationship and the potential evolutionary process.

### 2.2. Oil Red O Staining

**Figure 2:**
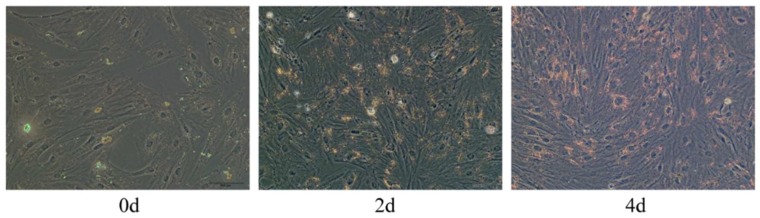

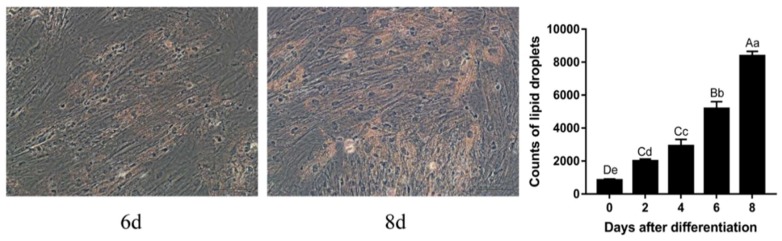
Oil Red O staining of differentiated adipocytes from Qinchuan cattle on different days following differentiation (Line 1: right to left, top to bottom: Day 0, Day 2, and Day 4 after differentiation, d: day; Line 2: Day 6 and Day 8 after differentiation; 200 μm), visible LDs detected two days after differentiation. Day 6 and Day 8 had larger quantities of LDs than Day 4 or Day 2. Different lowercase letters indicate significant differences (*p* < 0.05), and different capital letters indicate extremely significant differences (*p* < 0.01)

Oil Red O staining revealed that the amount of visible and sporadic lipid droplets increased starting from two days after differentiation (Figure 2). With increasing time after differentiation, the number of lipid droplets increased, indicating that adipocytes gradually differentiated and matured; at eight days after differentiation, many stained lipid droplets could be observed, indicating that, at eight days after differentiation, induced adipocytes were fully mature. This growing amount of lipid droplets was also measured by image J.

### 2.3. PLIN2 Expression and Location

**Figure 3:**
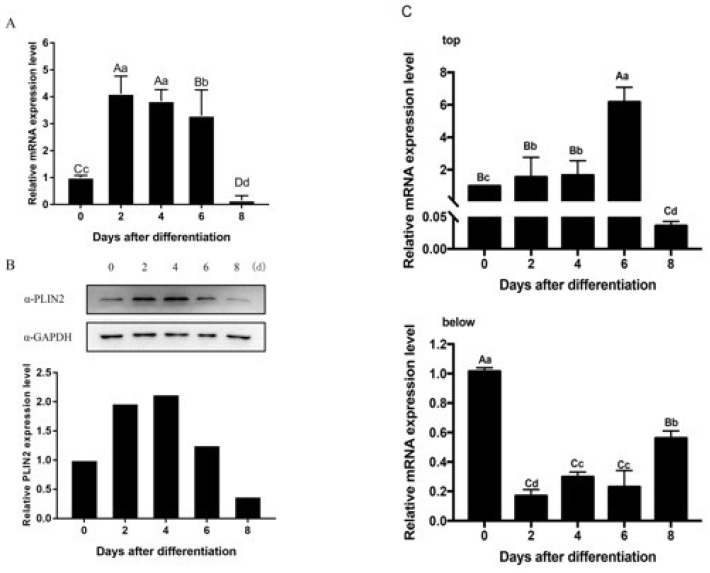
(**A**) PLIN2 gene expression levels in differentiated adipocytes from Qinchuan cattle using the qRT-PCR method. Different lowercase letters indicate significant differences (*p* < 0.05), and different capital letters indicate extremely significant differences (*p* < 0.01); (**B**) PLIN2 protein expression levels in differentiated adipocytes from Qinchuan cattle using the Western blot method. PLIN2 protein was recognized as a 47 kDa band, GAPDH (36 kDa) was used as a reference; (**C**) Genes related with adipocytes were measure as positive control (**Top**: PPARγ; **below**: CEBPα), d: day.

The PLIN2 mRNA expression pattern was determined in adipocytes after induction, using qRT-PCR (Figure 3A). The mRNA expression level of *plin2* peaked at two days after differentiation induction, and the expression level of the gene was significantly higher at Day 2 compared with Day 0 or with Day 8 (*p* < 0.01). From six days after differentiation to eight days after differentiation, the mRNA expression level decreased sharply, and the relative expression level on Day 8 after differentiation was only 15% of the level observed on Day 0. This result shows that the mRNA expression level of the gene changes with time.

**Figure 4:**
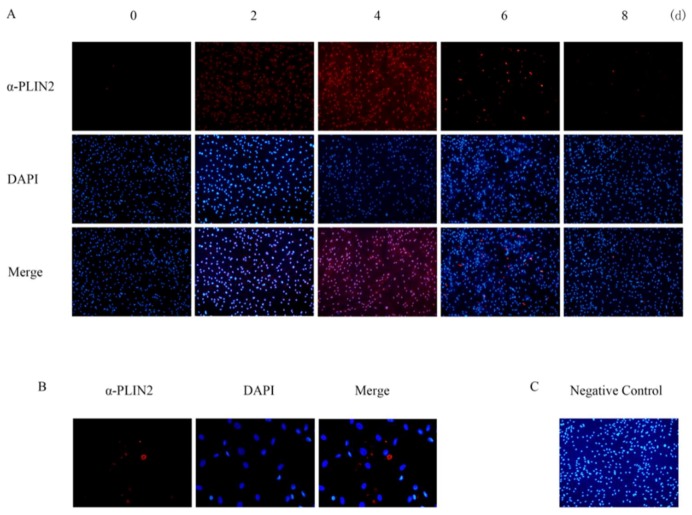
(**A**) Positive staining for PLIN2 (line 1 and line 3) is shown in red, while nucleus is shown in blue (right to left, top to bottom: Day 0, Day 2, Day 4, Day 6, and Day 8 after differentiation) (200 μm). (**B**) Matured lipid droplets can first be detected two days after differentiation (50 μm), d: day. (**C**) Negative control (200 μm).

The PLIN2 protein expression pattern is significantly different from the gene expression pattern, as assessed by Western blot analysis (Figure 3B). PLIN2 protein expression levels rose gradually and reached a maximal expression level four days after differentiation, then reduced sharply from six days after differentiation to eight days after differentiation. By the end of the experiment, the protein was nearly undetectable. Immunofluorescence assays detected PLIN2 protein during different stages of adipocyte differentiation. We confirmed that PLIN2 proteins anchored in the cytoplasm. Figure 3B and Figure 4 indicate that PLIN2 proteins were present in the cattle cells prior to adipocyte differentiation. Besides, we observed shaped LDs with PLIN2 antibody two days after differentiation; then, the quantity of PLIN2 increased gradually, and it appeared to be more ubiquitously diffuse at four days after differentiation. Consequently, it decreased starting at four days after differentiation and nearly vanished by eight days after differentiation, when many LDs had matured. Based on LD staining results, this evidence strongly suggests that PLIN2 is functional periodically, and when the lipid droplets mature, the protein production is decreased, leading us to consider whether the primary function of PLIN2 is to influence early LD formation. Some genes which expressed in adipocytes differentiation were also measured as positive control; in this paper, we showed PPARγ and CEBPα’s expression (Figure 3C).

### 2.4. Protein Structure Analysis

**Figure 5:**
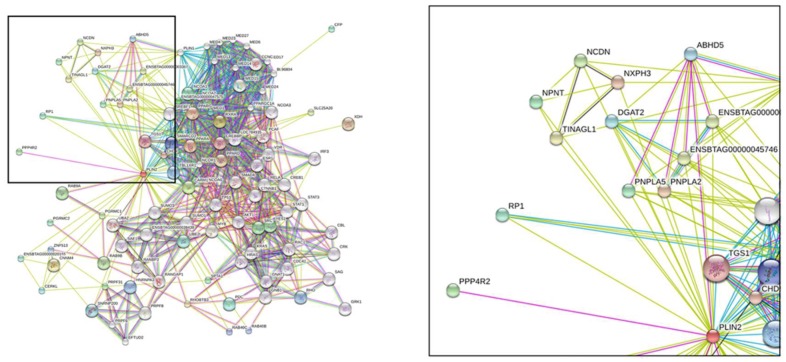
STRING analysis of bovine PLIN2 with interacting proteins, such as ABHD5 and PNPLAs.

**Figure 6:**
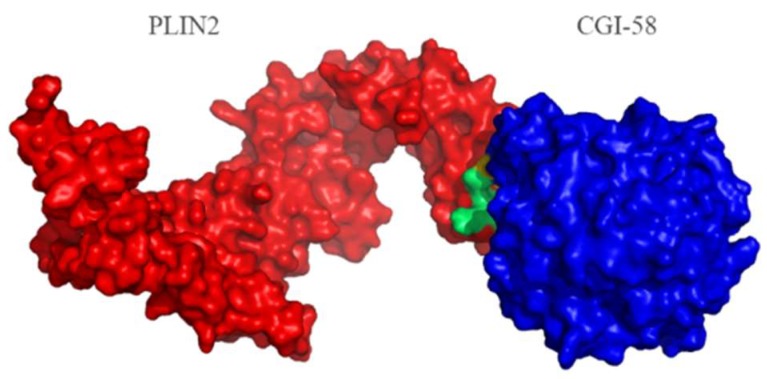
Interaction location analysis based on bovine PLIN2 and CGI-58 protein structures. The red section is the predicted PLIN2 model, the blue section is the predicted CGI-58 model, and the green section is the PLIN2-CGI-58 interface, which is located on PLIN2 (PRISM2.0 and PyMOL).

The PSIPRED [25] result indicated that PLIN2 is primarily formed by α-helices and one beta-sheet (Figure S1). The α-helix is the primary structure of membrane proteins because an α-helix has a prominent hydrophobic interaction function, and a beta-sheet structure can also pass through the membrane [26]. According to STRING analysis, we found that PLIN2 interacts with CGI-58 (ABHD5) (Figure 5). We then built a PLIN2 and CGI-58 interacting model using PyMOL software (Figure 6). We also used PRISM [27] to predict the likely interaction locus (shown in Table 1), which consisted of 19 amino acids that contribute to the PLIN2 interface. These results indicate that these amino acid residues play vital roles in the interaction between PLIN2 and CGI-58.

### 2.5. PLIN2 Interaction with CGI-58 Pull-Down Assay

**Figure 7:**
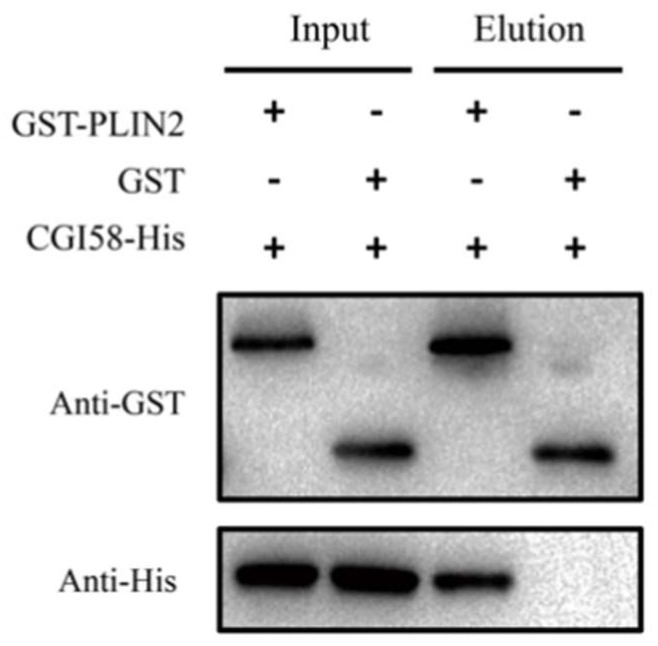
Binding of PLIN2 with CGI-58 upon GST-pull-down assay, in vitro. GST-fusion proteins containing the residues of full-length PLIN2 as bait and was incubated with the full-length His-CGI-58 (Input). After washing, input and bound proteins were analyzed by Western blotting with anti-His antibody (WB). Detection of CGI-58-His in the elution demonstrated that CGI-58-His could be captured with GST-PLIN2 fusion proteins in GST pull-down assays, verifying the interaction between PLIN2 and CGI-58.

Next, we used a GST (Glutathione S-Transferase) pull-down assay to verify the interaction between PLIN2 and CGI-58 in vitro. We expressed recombinant, GST-tagged, bovine PLIN2 and recombinant, His-tag, bovine CGI-58 in bacteria. Figure 7 shows that the full length CGI-58 was bound to PLIN2. This result supports the interaction relationship that was identified by the bioinformatics prediction model and indicate that PLIN2 and CGI-58 cooperate to regulate the early formation of lipid droplets during adipocyte differentiation.

### 2.6. Bimolecular Fluorescence Complementation with Flow Cytometry (BiFC-FC)

**Figure 8:**
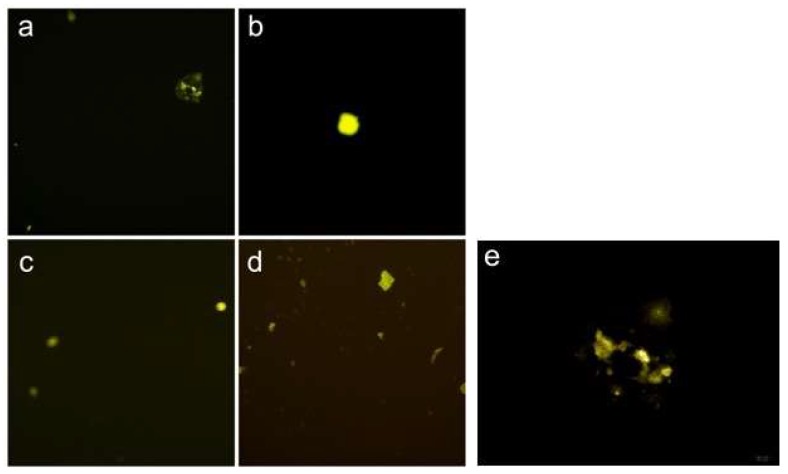
Visualization of PLIN2 and CGI-58 interaction groups in bovine adipocytes. The fluorescence emissions of the cells were imaged 24 h after transfection: (**a**) PLIN2-VC173+CGI-58-VC155; (**b**) PLIN2-VN173+PLIN2-VC155; (**c**) CGI-58-VN173+CGI-58-VC155; and (**d**) CGI-58-VN173+PLIN2-VC155. The strong fluorescence of Venus in cells co-expressing CGI-58-VN173 and PLIN2-VC155, detected by fluorescence microscope, demonstrated that CGI-58 could interact with PLIN2. Weak fluorescence in other cells was also detected. These results revealed the interacting relationships between PLIN2 and CGI-58 in vivo, including self-interaction (OLMPUS. 100 μm); (**e**) PLIN2-VC173+CGI-58-VC155(OLMPUS. 50 μm).

**Figure 9:**
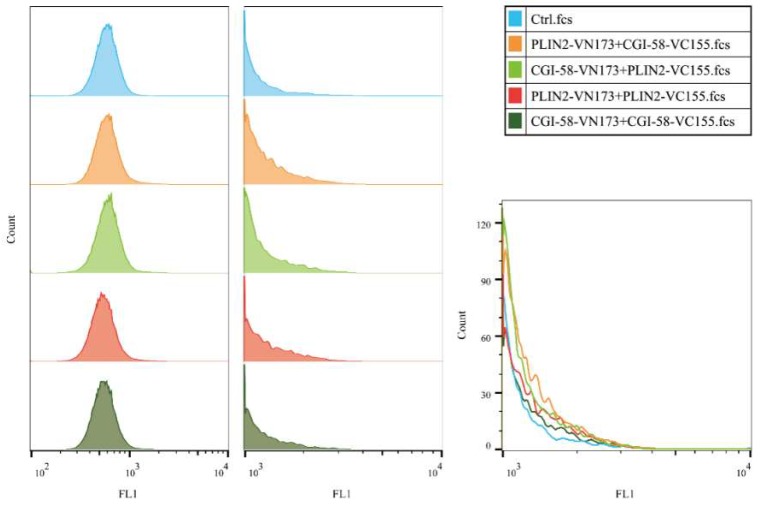
Flow cytometry showed that all samples harbored stronger fluorescence intensity than control cells (bovine adipocytes), although the number of detected cells containing fluorescence were not abundant (**Left**). The fluorescence intensity of cells containing PLIN2-VN173 and CGI-58-VC155 was stronger than cells harboring other protein–protein pairs. Cells harboring higher fluorescence intensity were gated (**Middle**), and the Counts-Fluorescence intensity distribution of these cells were analyzed by FlowJo^®^ software (**Right**).

The Bimolecular Fluorescence Complementation (BiFC) assay has been widely used to detect protein–protein interactions (PPIs) in living cells [28,29]. This technique is based on the reconstitution of a fluorescent protein in vivo. We used this assay to determine the PPI relationship between PLIN2 and CGI-58 by constructing different vectors (Table 2) that were expressed in bovine adipocytes. The results showed that PLIN2-VN173+CGI-58-VC155 and CGI-58-VN173+PLIN2-VC155 both displayed fluorescence with different intensities, a phenomenon that suggested that different protein–protein interaction structure models might influence the protein–protein interaction efficiency. PLIN2-VN173+PLIN2-VC155 showed fluorescence with a low intensity (Figure 8), and CGI-58-VN173+CGI-58-VC155 also showed Venus fluorescence, which suggests that PLIN2 and CGI-58 might self-interact to regulate LD formation. Moreover, we detected the fluorescence intensity of each group through Flow Cytometry (Figure 9). Although there were no significant differences in fluorescence among the groups, FCM showed that PLIN2-VN173+CGI-58-VC155 had the highest interaction efficiency. These PPI results lead us to consider that the function of PLIN2 may require cooperation with CGI-58 for early lipid droplet formation. We also performed the BiFC-FC experiment in 3T3-L1 cells (Figures S2 and S3), but the fluorescence expressed differently compared with bovine adipocytes, with CGI-58-VN173+PLIN2-VC155 demonstrating the highest efficiency in 3T3-LI cells.

### 2.7. Amino Acid Mutation Analysis 

**Figure 10:**
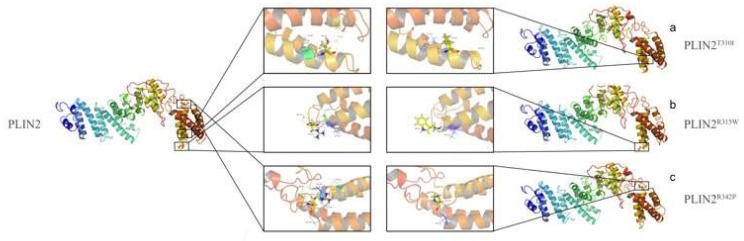
Amino acid structure of bovine WT PLIN2 and the structures of three mutation-variants of PLIN2 (T310I, R315W, R342P) (I-TASSER): (**a**) threonine at position 301 mutated to isoleucine, which influenced the interaction with 307E and 375K (on the interface); (**b**) arginine at position 315 on the interface mutated to tryptophan, which influences the interaction with 316N; and (**c**) arginine at position 342 mutated to proline, which influence interaction with 298D, 299E, 331N, and 338N.

In our previous study, five SNP loci were detected in *Plin2* that were associated with beef cattle meat-quality traits. Later, we used bio-software to analyze these SNPs and their effects on the protein tertiary structure (Figure 10). T310I (g.C7919>T) showed the structure combination changing with 307GLU and 375LYS. Notably, ARG315TRP (g.C7933>T) is on the interface where PLIN2 interacts with CGI-58. An interaction between R315 and 316ASN, which is associated with IMF content (Intramuscular Fat) and BF thickness (Back Fat), is disrupted by the R315W mutation. ARG342PRO (g.G8015>C) also influenced the tertiary structure with 298ASP, 299GLU, 331ASN, and 338ASN. These results lead us to hypothesize that these loci are key sites for the function of PLIN2 and for the interaction between PLIN2 and CGI-58. Further study will be performed to verify the functions of these amino acids and *Plin2* gene’s functional research for lipid metabolism.

## 3. Discussion

Research on PLIN2 has primarily examined the protein’s function, early LD formation, and energy metabolism, and it appeared that this protein prefers to localize to small droplets [30,31]. Recent studies have shown that PLIN2-deficient mice, without any truncated protein products, can be protected against adipose inflammation [32]. We detected PLIN2 expression in cattle adipocytes, and, then, we aimed on determine the relationship between mRNA and protein expression. The peak mRNA and protein expression patterns are not synchronous. It is known that PLIN2 can coat on small LDs in preadipocytes and function is associated with lipid formation in early stage of adipocytes differentiation; besides, when the cells are differentiated into mature adipocytes, PLIN2 disappears or is completely displaced by perilipin1 on the surface of matured lipid droplets [15,33]. Our research on induced differentiated adipocytes from cattle demonstrates that mRNA level peaked at two days after differentiation, however protein level reached maximum at four days after differentiation. In addition, the protein still exists during the differentiation stage and a large quantity of protein can be detected six days after differentiation. Interestingly, the Oil Red O staining results show that the LD quantity increased and that the LD shape enlarged gradually, but PLIN2 protein levels decreased six days after the induction of adipocyte differentiation. We speculated that: (a) modifications, such as methylation or acetylation, may influence the quantity of protein production, and we do not clearly understand the whole mechanisms of protein processing and assembly; and (b) some regulatory systems exist to control PLIN2 degradation, such as the ubiquitin-proteasome system, which plays a critical role in protein quality control and protein degradation in eukaryotic cells [34,35]. Many proteins are subjected to the system and its dysregulation is linked to the pathogenesis of diseases, such as cancer and neurodegeneration [36]. PLIN2 has also been reported to be a substrate of the 26S proteasome [19,37]. Thus, we considered how PLIN2 expression levels decrease and whether a special regulatory mechanism exists via the ubiquitin-proteasome system.

It is necessary to use bioinformatic methods to analyze the potential protein structure. Collectively, we obtained PLIN2′s structure and interaction sites and these data will provide supporting evidence for future studies on PLIN2. A mutational analysis revealed that the ubiquitination and degradation of the protein required both the second and third alanine in the N-terminal region, which follows the N-terminal rule [38,39]. Protein structure modeling shows amino acid mutation influences the ability of the protein to bind with LDs, which has an important role in early lipid formation, but the protein structure and function remain largely unknown. Two studies have shown that PLIN1 and PLIN2 can both combine with CGI-58, which is a critical regulator of the initial process of combining the LD surface membrane and lipolytic enzymes. The glycerol release was decreased by *Plin2* knockdown after 4 and 8 h of the lipolytic stimulation. These results also demonstrated that *Plin2* increased lipolysis and did not protect LDs from the lipolytic enzyme attacks in MEFs [3,40]. Furthermore, CGI-58 plays a vital role in TAG storage in humans via linking with proteins. ATGL (adipose triglyceride lipase, also known as PNPLA2) functions as a critical lipase with specificity toward TAG. CGI-58 has been shown to indirectly promote TAG turnover by serving as a co-activator of ATGL. A disease named NLSD was caused by CGI-58 mutations which leads to CGI-58 function lost. Further research will focus on figuring out the lipolytic rate-limiting enzymes and their regulation mechanism [3,41]. In addition, we used GST-pull-down assays to confirm that PLIN2 and CGI-58 have an interacting relationship. BiFC-FC methods were used to determine the exact interaction relationship, and the results demonstrated that different PLIN2-CGI-58 interaction models resulted in different interacting efficiencies. These differences might be explained by the idea that a diverse protein binding environment can influence the protein interspace structure, which plays a vital role in protein function. Of note, we obtained discrepant results when we performed the same experiments in bovine adipocytes (Table 2.) and in 3T3-L1 cell lines (Table S2). This might be caused by the different intracellular environment in 3T3-L1 cells because they have several distinct characteristics compared with bovine adipocytes. For example, SREBP-1c is not expressed in 3T3-L1 adipocytes but is expressed in bovine adipocytes, in vivo. Consequently, different fluorescence efficiencies may be obtained. Our bio-information research predicted that the bovine PLIN2-CGI-58 binding interface is on the C-terminal region. Amino acids, such as the Arginine at site 315, might be the key loci for interaction, because Arginine’s side chain groups are positively charged and easily interact with the side chains of other amino acids to form binding sites [26]. Future research will be performed to identify the key amino acids on the interface via mutation experiments. Another member of the PAT family, PLIN1, requires the phosphorylation of one or more serines within the three-amino-acid terminal PKA site facilitate hormone-sensitive lipase access to lipid substrates [42]. When catecholamines bind to cell surface receptors to initiate signals that activate cAMP-dependent protein kinase (PKA), phosphorylated PLIN1 facilitates maximal lipolysis. This evidence leads us to consider that modifications may also influence PLIN2′s function. According to STRING analysis, we also found the following factors: (a) PPARA is vital for binding with PLIN2. PPARA is also involved in lipid metabolism and carbohydrate metabolism [43]. (b) ATGL(PNPLA2) is the rate-limiting lipolytic enzyme of TG breakdown. Future studies can focus on verifying the PPARA–PLIN2 and ATGL–PLIN2 interaction relationships and their function on regulating lipid metabolism. This common combination of proteins led us to consider whether there is a regulation system that influences the “balance” or “replacement” between PLIN1 and PLIN2. Another study shows that PLIN2 also has a lipolysis function [20], and the results showed lentiviral-mediated *Plin2* knockdown attenuated lipolysis in differentiated MEFs in a time-dependent manner. Moreover, oleic acid-induced lipid droplets formation enhanced PLIN2 stability when it was localized to lipid droplets. Our research provides further evidence of the PLIN2 interaction relationship and suggests a deeper comprehensive regulation mechanism. We hypothesize that this might be a crucial reason for PLIN2′s lipolysis role, and its basic function might be complicated by interactions with other partners.

In summary, we demonstrated PLIN2′s expression patterns in cattle adipose tissue and in differentiated adipocytes and locations within adipocytes; these findings provide evidence for understanding cattle adipocytes LDs formation. Some studies have been reported in rats and humans and in different pathologic conditions [43,44], but no studies have been performed in cattle, thus far. Future study is needed to discern the molecular regulating mechanism of PLIN2 during LD formation and development. By determining the functions of PLIN2, the whole network of lipid metabolism, which is a pivotal physiological response essential for systemic energy homeostasis, becomes much clearer. 

## 4. Material and Methods

All animal procedures were performed according to guidelines set by Regulations for the Administration of Affairs Concerning Experimental Animals (Ministry of Science and Technology, China, 2004). Animals were fed the same feed of roughage at a concentrate ratio of 6:4 on a total mixed ration (TMR) basis and approved by the Institutional Animal Care and Use Committee (EAMC protocols, College of Animal Science and Technology, Northwest A&F University, China, No.2013-23, 20 April 2013). 

A three-day-old healthy Qinchuan beef cattle was used for adipocytes isolation and cell culture. It was born and raised at the experimental farm of National Beef Cattle Improvement Center (Yangling, China) and slaughtered using mechanized slaughter line at Shaanxi Qinbao Animal Husbandry Development Co., Ltd. (Xi’an, China). 

### 4.1. Isolation and Cell Culture of Bovine Adipocytes

3T3-L1 pre-adipocytes were cultured in Dulbecco’s modified Eagle’s medium (Gibco, Grand Island, NY, USA), containing 4 mM glutamine, 1 mM sodium pyruvate, 10% fetal bovine serum, 1% penicillin, and 1% streptomycin (Gibco).

Bovine adipocyte collection Pre-adipocytes were separated from Qinchuan fetal bovine samples using a protocol supplied by the National Beef Cattle Improvement Center. The adipocytes tissues sample was obtained from slaughter beef cattle. The subcutaneous skin was rinsed with 75% ethanol and removed with sterile sharp curved surgical scissors to expose the adipocytes tissue. The proper amount of subcutaneous adipocytes tissues was then removed into 1 × PBS supplemented with 10% penicillin/streptomycin and was immediately taken into the cell culture lab. Under a stereo dissecting microscope, the adipocytes sample was dissected away from the blood vessel and connective tissue with sterile forceps. The adipocytes tissue was then minced and digested with 0.25% Collagenase I (Sigma, Kawasaki City, Japan)/0.1% Diapase II (Roche, Basel, Switzerland) solution at 37 °C until the mixture is a fine slurry. The cell suspension was filtered through 80-μm cell strainer and pelleted by centrifugation for 5 min at 350× *g*. The pellets were then resuspended and seeded in 60-mm collagen-coated culture plates followed by purification procedure. The primary adipocytes were cultured in complete growth medium containing Dulbecco’s modified Eagle’s medium/F-12 (DMEM/F-12, Gibco), 20% fetal bovine serum (Gibco) and 1% penicillin/streptomycin. Growth medium was changed every two days and passaged at 70% confluence to avoid spontaneous differentiation.

Pre-adipocytes were cultured in Dulbecco’s modified Eagle’s medium (DMEM) with 10% fetal bovine serum (FBS), 1% penicillin, and 1% streptomycin (Gibco). 

### 4.2. Induction of Adipocytes Lipid Droplet Formation

3T3-L1 cells and bovine adipocytes were induced, following the induction protocol, with isobutylmethylxanthine (IBMX, 0.5 mM), insulin (1 mg/mL), and Dex (Sigma 1 mM, Saint Louis, MO, USA). The first day of induction was defined as Day 0. Then, adipocytes were cultured with 100 nM insulin alone until adipocyte differentiation was complete (Days 2–8), and culture solution were renewed every two days. All culture systems were performed at 37 °C in 95% humidity with 5% CO_2_ (Thermo Fisher Scientific, Waltham, MA, USA).

### 4.3. Oil Red O Staining and Counting Lipid Droplets

Adipocytes were fixed with 4% paraformaldehyde in PBS for 1 h at room temperature and then stained with Oil Red O solution (0.5% Oil red O in 2-propanol: milliQ = 3:2 (*v*/*v*)) for 1 h at room temperature. Average size of 100 lipid droplets per sample was measured on Image J software (version 1.47 v; NIH, Bethesda, MD, USA) with adjusting each picture’s background. Each visible oil red O stained droplet was manually traced using the circle tool of Image J software, which recorded the diameter of each droplet. 

### 4.4. Quantitative RT-PCR

Total cellular RNA was extracted from bovine adipocytes using TRIZol (Takara, Mountain View, CA, USA), and reverse transcription was performed, it was then applied to synthesize cDNA using PrimeScript™ RT reagent Kit with gDNA Eraser (Takara). The reverse transcript reaction was using cDNA Reverse Transcription Kit (Takara) and performed at 37 °C for 15 min followed by 85 °C for 5 s. mRNA expression levels were determined using real-time PCR on an ABI PRISM 7500 (Applied Biosystems, Foster City, CA, USA) using a SYBR^®^ Kit (Takara). GAPDH was used as an internal control to normalize target gene mRNA levels. Real time PCR was performed with 3 repeats (sequence in Table S1). The data were analyzed with the 2^−∆∆*C*t^ method [45].

### 4.5. Western Blot Analysis

Cell proteins were extracted with a protein extraction Kit (Solarbio Company, Beijing, China), then mixed with protein loading buffer and denatured at 100 °C for 10 min with 1 mM phenylmethylsulfonyl fluoride (Millipore, Billerica, MA, USA), and 1 mM ethylenediaminetetraacetic acid (EDTA). The membranes were then incubated sequentially with antibodies against GAPDH (rabbit anti-GAPDH, 1:10,000 Abcam, Cambridge, UK) and PLIN2 (rabbit anti-PLIN2, 1:800 Abcam NT, HK) for 12 h at 4 °C. After washing three times (10 min each) with PBS-Tween 20, the membranes were incubated with secondary IgG-Goat anti-Rabbit HRP antibodies (1:2000 NOVUS NT, HK) diluted in PBS-Tween 20 (0.08 μg/mL) for 2 h, and then washed in PBS-Tween 20. Chemiluminescent detection (Millipore) was performed by mixing equal volumes of Luminol Reagent and Peroxide solution in a clean container or test tube and then applying the mixture to PVDF membranes. Immunoreactivity was detected using a Gel Doc™ XR+ Gel Documentation System (Bio-Rad, Hercules, CA, USA).

### 4.6. Immunofluorescence

Day 0, 2, 4, 6, and 8 adipocytes were cultured on six-well culture plates and fixed with 4% paraformaldehyde for 15 min, washed with PBS fixed in 4% paraformaldehyde for 15 min at room temperature, then washed in PBS three times (HyClone, Logan, UT, USA). Adipocytes were treated with 1% Triton X-100 (Sigma), then washed in PBS buffer three times and blocked for 30 min in a 1% BSA (Sigma) solution. Samples were incubated with primary Rabbit anti-PLIN2 antibody (Abcam, 1:500 dilution in 1% BSA/10% donkey serum/0.3 M glycine) overnight at 4 °C. After washing with PBS, adipocytes were incubated with the second antibody (1:1000 dilution in 1% DSA solution for 1 h, protected from light at 37 °C), donkey anti-rabbit IgG H&L (Alexa Fluor^®^555, Abcam, Cambridge, UK), then 1 µg/mL DAPI (Sigma) was added and incubated for 10 min. A negative control was used for each secondary antibody, using adipocytes that were not incubated with primary antibody. DAPI was used at the final concentration of 1 μg/mL. Different stages of adipocytes were captured and analyzed using a fluorescent microscope (OLMPUS IX71, OLMPUS, Tokyo, Japan).

### 4.7. Bioinformatic Study

The amino acid sequences of PLIN2 in different species (*Bos Taurus*, *Homo sapiens*, *Rattus norvegicus*, *Mus musculus*, *Capra hircus*, *Ovis aries* and *Gallus gallus*) were BLASTed by NCBI. A phylogenetic tree for PLIN2 was built using MEGA v6.06. A multiple sequence alignment for orthologous PLIN2 proteins was performed using Clustal Omega (http://www.clustal.org/clustal2/). Secondary structure feature prediction for PLIN2 was calculated by PSIPRED v3.3 (Predict Secondary Structure http://bioinf.cs.ucl.ac.uk/psipred). The spatial structure of PLIN2 was predicted by the I-TASSER (Iterative Threading ASSEmbly Refinement) [46,47] server online tool (http://zhanglab.ccmb.med.umich.edu/I-TASSER). The PLIN2-CGI-58 (ABHD5) interaction model was predicted by PRISM 2.0 (http://cosbi.ku.edu.tr/prism/index.php). The structure files were analyzed using PyMOL v1.7.4.5 Edu. The impact on protein biological function due to several amino acid mutations was predicted using PROVEAN v1.1.3 (Protein Variation Effect Analyzer http://provean.jcvi.org/seq_submit.php). The functional protein association network was analyzed by STRING v10.5 (http://string-db.org).

### 4.8. GST Pull-Down Assays

To construct expression plasmids for CGI-58 and PLIN2, cDNA fragments were generated by PCR with appropriate primers (Table S3). Fragments were subcloned into pGEX-2T-PLIN2 (GE Healthcare Little Chalfont, Buckinghamshire, UK) and pET-28-CGI-58-His (Novagen, Kenilworth, NJ, USA) vectors with ClonExpress II One Step Cloning Kit (Vazyme, Nanjing, Jiangsu, China) and transfected into *E. coli* BL21 (DE3). The expression of recombinant glutathione *S*-transferase (GST)-PLIN2 fusion proteins was induced by IPTG (final concentration of 0.2 mM) at 16 °C for 16 h, while CGI-58-His chimeras were expressed at 4 °C for two days, supplemented with 0.5 mM IPTG. The *E. coli* cells were disrupted by sonication. GST-PLIN2 fusion proteins were purified using Glutathione Sepharose™ 4B (GE Healthcare) and were eluted by phosphate buffer (137 mM NaCl, 2.7 mM KCl, 10 mM Na_2_HPO_4_, and 2 mM KH_2_PO_4_ (pH 8.0)). The lysates containing overexpressed CGI-58-His were extracted by a protein extraction Kit (Solarbio Company). 

Purified GST-PLIN2 fusion proteins were mixed with lysates expressing CGI-58-His at 4 °C for 2 h. GST incubating with the above-described lysates served as the negative control. Pull-down assays were performed using Glutathione Sepharose™ 4B (GE Healthcare) in 1.5 mL tubes. After washing with PBS (140 mM NaCl, 2.7 mM KCl, 10 mM Na_2_HPO_4_, 1.8 mM KH_2_PO_4_), proteins bound with GST-PLIN2 or GST were eluted by elution buffer (10 mM Glutathione in 50 mM Tris-HCl, pH 8.0) and collected. Next, the interaction between GST-PLIN-2 and CGI-58-His was detected by SDS-PAGE and analyzed by Western blotting. Probing with a GST-specific antibody (CW0291M, CoWin Biosciences, Beijing, China), PLIN2 was detected, and CGI-58 was recognized by a His-tag-specific antibody (CW0286M, CoWin Biosciences). The secondary antibody was a goat anti-mouse antibody (CW0110, CoWin Biosciences).

After centrifugation at 10,000× *g* for 30 min at 4 °C, the resulting supernatant was used to bind with Glutathione-Sepharose beads containing 15 µg of different GST-fused proteins. Beads were washed using ice-cold binding buffer, after a 4 h incubation at 4 °C, and resuspended in 100 µL of SDS-PAGE loading buffer. A 20 µL aliquot was separated by SDS-PAGE and analyzed by Western blotting with anti-GST and anti-His monoclonal antibodies (CoWin Biosciences).

### 4.9. Bimolecular Fluorescence Complementation (BiFC)

This assay allows for the rapid visualization of the compartment-specific interactions of a protein complex, and PPIs can be easily quantified in vivo. BiFC expression plasmids for bovine PLIN2 and CGI-58 were similarly constructed by inserting the PCR fragment containing full length PLIN2 or CGI-58 (primer in Table S4) into pBiFC-VN173 and pBiFC-VC155 (Addgene, Cambridge, MA, USA) with ClonExpress II One Step Cloning Kit, named as PLIN2-VN173, PLIN2-VC155, CGI-58-VN173, and CGI-58-VC155. Bovine adipocytes were grown in six-well plates to 80% confluence in10% fetal bovine serum (FBS), 1% penicillin, and 1% streptomycin at 37 °C in 95% humidity with 5% CO_2_. These plasmids were transfected into bovine adipocytes using Lipofectamine^®^ 3000 (Invitrogen, Carlsbad, CA, USA). Fluorescence could be detected after 24 h by microscope (OLMPUS IX71).

### 4.10. Flow Cytometry

Bovine adipocytes and 3T3-L1 cells were harvested 24 h following co-transfection by centrifugation at 300× *g* for 1 min. PBS was used to resuspend the pellet. Cell detection was performed on a CyFlow Cube 6 flow cytometer (PARTEC Gorlitz, Saxony, Germany) at an excitation wavelength of 488 nm. Cells harboring post-assembly Venus fusion proteins were detected in the FL1 channel at an emission wavelength of 530 nm. Data were collected from 8 × 10^4^ cells for each sample and, cell populations were analyzed offline using FlowJo^®^ software (v.10.5, BD, Franklin Lakes, NJ, USA). 

### 4.11. Statistical Analysis

All data are presented as mean ± SEM. Statistically significant differences between two groups were analyzed using Independent-samples *t*-test, and among three or more groups were analyzed by SPSS (version 23.0, IBM, Armonk, NY, USA) in ANOVA.

## Figures and Tables

**Table 1:** Bovine PLIN2 predicted binding sites with CGI-58.

CGI-58		PLIN2
TRP_27	<-->	LEU_371
PHE_119	<-->	SER_367
TRP_27	<-->	LYS_375
PHE_119	<-->	LYS_364
PHE_119	<-->	GLU_365
CYS_28	<-->	GLN_379
PHE_119	<-->	ASP_368
GLU_259	<-->	SER_367
TRP_27	<-->	ALA_314
ARG_118	<-->	ASP_368
TRP_27	<-->	ILE_313
ARG_118	<-->	SER_367
PRO_25	<-->	GLN_379
ARG_118	<-->	GLY_369
THR_26	<-->	GLY_376
THR_26	<-->	LYS_375
THR_255	<-->	ARG_315 *
THR_255	<-->	ALA_314
PRO_29	<-->	LEU_311

* indicates an amino acid mutation site caused by an SNP (g.C7933>T) in PLIN2.

**Table 2:** BiFC results with different PLIN2 and CGI-58 vectors in bovine adipocytes.

Vector Names	Efficiency
PLIN2-VN173+CGI-58-VC155	++
PLIN2-VN173+PLIN2-VC155	+
CGI-58-VN173+CGI-58-VC155	+
CGI-58-VN173+PLIN2-VC155	++

+: low fluorescence intensity; ++:high fluorescence intensity.

## Supplementary Materials

Supplementary materials can be found at http://www.mdpi.com/1422-0067/19/5/1336/s1.
