# Supplementary material for: The Expression Pattern of PLIN2 in Differentiated Adipocytes from Qinchuan Cattle Analysis of Its Protein Structure and Interaction with CGI-58
Source: Int J Mol Sci. 2018 May 1;19(5):1336. doi: 10.3390/ijms19051336 (PMC5983586; doi:10.3390/ijms19051336)
Supplement: Supplementary file 1 [file ijms-19-01336-s001.zip › ijms-292385-SI.pdf]

Supplementary Materials

Table S1. Primer Sequence.

Gene Name	Primer Sequence/(5′→3′)	Annealing Temperature/°C	Product Size/bp
PLIN2	F: CTGTCTACCAAGCTCTGCTC<br>R: CGATGCTTCTCTTCCACTCC	80.2	120
GAPDH	F: ACCACAGTCCATGCCATCAC<br>R: TCCACCACCCTGTTGCTTA	84.9	124

Table S2. BiFC results with different PLIN2 and CGI-58 vector in 3T3-L1 cell lines.

Vectors Name	Efficiency
PLIN2-VN173+CGI-58-VC155	++
PLIN2-VN173+PLIN2-VC155	++
CGI-58-VN173+CGI-58-VC155	+
CGI-58-VN173+PLIN2-VC155	+

Table S3. GST-pulldown vector of PLIN2 and CGI-58 primers.

Gene Name	Primer Sequence/(5′→3′)
GST-PLIN2	F: gatctggttccgcgtggatccGCATCTGTTGCAGTTGAACCAC<br>R: tcagtcagtcacgatgaattcTCAAGAGGAGCTGTCATCTGGC
HIS-CGI-58	F: atcaaaggagatataccatggCGGCAGAGGAGGATGGGG<br>R: atggtgatggtggtgctcgagGTCCACAGTGTCACAGATCTCC

Table S4. BiFC vector of PLIN2 and CGI-58 primers.

Gene Name	Primer Sequence/(5′→3′)
VN-173-PLIN2	F: caagcttgcggccgcgaattcAGCATCTGTTGCAGTTGAACCA<br>R: ggtggcgatggatcttctagaAGAGGAGCTGTCATCTGGCTGG
VN-173-CGI-58	F: caagcttgcggccgcgaattcAGCGGCAGAGGAGGATGG<br>R: ggtggcgatggatcttctagaGTCCACAGTGTCACAGATCTCCTTC
VC-155-PLIN2	F: tggccatggaggcccgaattcAGGCATCTGTTGCAGTTGAACC
	R: acgccggacgggtacctcgagAAGAGGAGCTGTCATCTGGCTG
VC-155-CGI-58	F: tggccatggaggcccgaattcAGGCGGCAGAGGAGGATG
	R: acgccggacgggtacctcgagAGTCCACAGTGTCACAGATCTCCTT

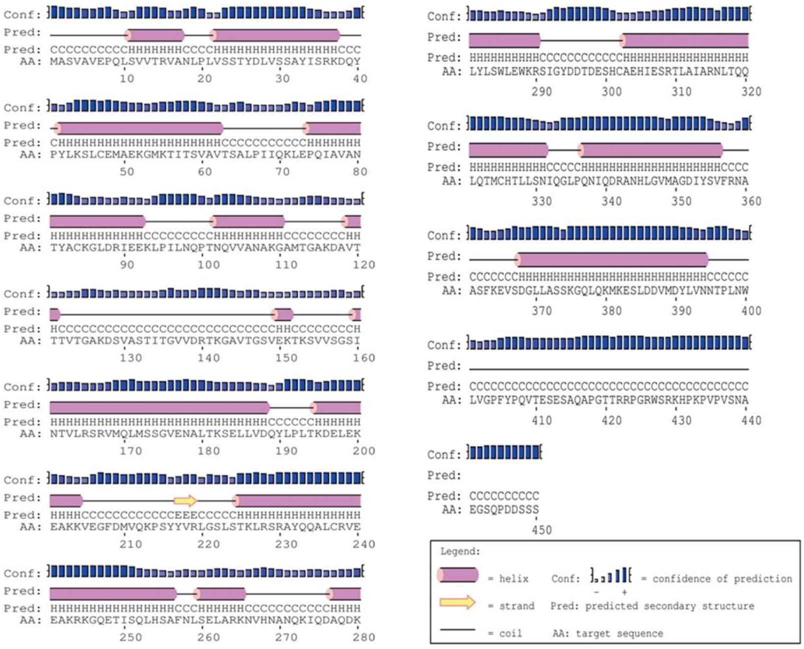

Figure S1. visualization of PLIN2 secondary structure of helix, strand and coil. It can be indicated PLIN2 contains helix mainly after prediction.

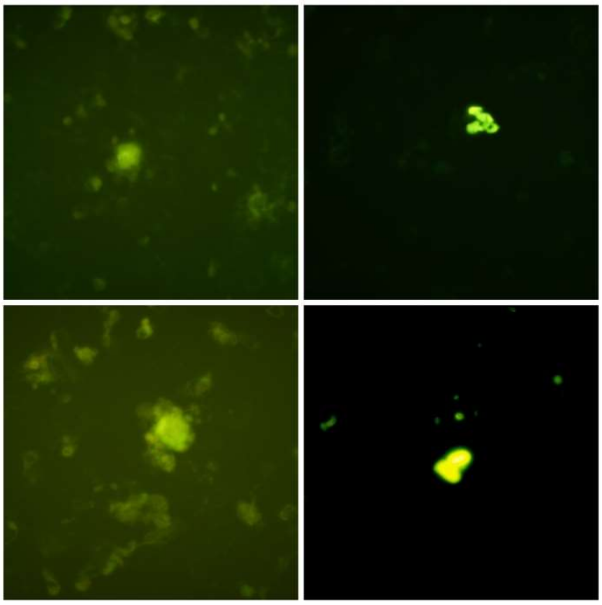

Figure S2. visualization of PLIN2 with CGI-58 interaction groups in bovine adipocytes, the fluorescence emissions of the cells were imaged 24h after transfection(a, PLIN2-VC173+CGI-58-VC155, b, PLIN2-VN173+PLIN2-VC155(first line right), c, CGI-58-VN173+CGI-58-VC155, d, CGI-58-VN173+PLIN2-VC155). The strong fluorescence of Venus in cells co-expressing CGI-58-VN173 and PLIN2-VC155, CGI-58-VN173 and CGI-58-VC155 detected by fluorescence microscope demonstrated CGI-58 could interact with PLIN2. The weak fluorescence in other cells were also detected. These results revealed the interacting relationships between PLIN2 and CGI-58 in vivo, including the self-interaction (OLMPUS 200X).

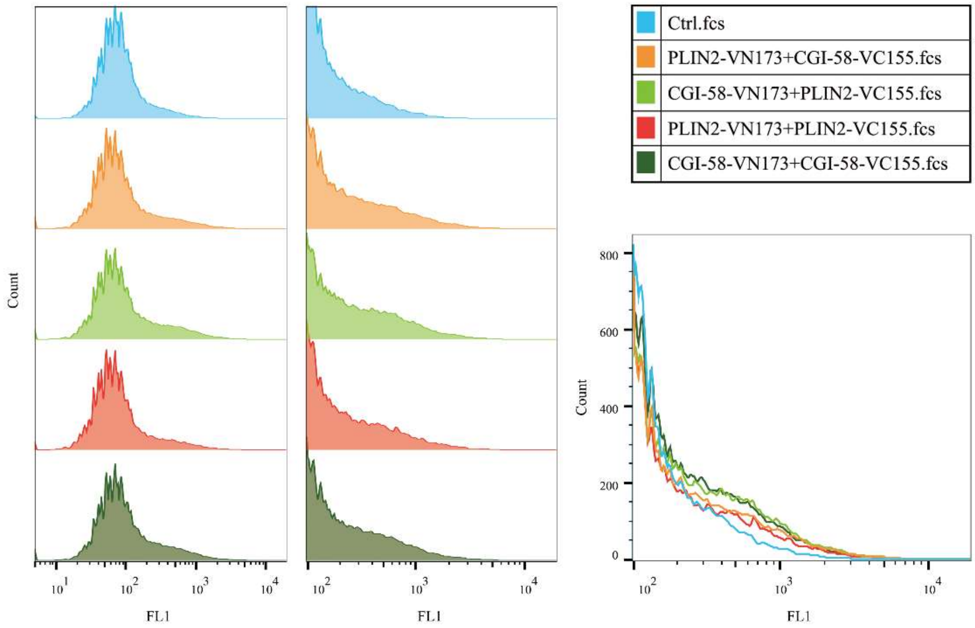

Figure S3. Flow cytometry showed all samples harbored the stronger fluorescenceintensity that control cells (3T3-L1 cells), although the number of detected cells containing fluorescence are not abundant (**Left**). A noticed data that the fluorescenceintensity of cells containing CGI-58-VN173 and PLIN2-VC155, CGI-58-VN173 and CGI-58-VC155 were stronger than cells harboring other protein-protein pairs. Cells harboring higher fluorescenceintensity were gated (**Middle**) and then the Counts-Fluorescenceintensity distribution of these cells were analyzed by FlowJo® software (**Right**).
